# Gene expression profiling of liver from dairy cows treated intra-mammary with lipopolysaccharide

**DOI:** 10.1186/1471-2164-9-443

**Published:** 2008-09-24

**Authors:** Li Jiang, Peter Sørensen, Christine Røntved, Lotte Vels, Klaus L Ingvartsen

**Affiliations:** 1 Department of Genetics and Biotechnology, Faculty of Agricultural Sciences, University of Aarhus, DK-8830 Tjele, Denmark; 2 Department of Animal Health, Welfare and Nutrition, Faculty of Agricultural Sciences, University of Aarhus, DK-8830 Tjele, Denmark

## Abstract

**Background:**

Liver plays a profound role in the acute phase response (APR) observed in the early phase of acute bovine mastitis caused by *Escherichia coli *(*E. coli*). To gain an insight into the genes and pathways involved in hepatic APR of dairy cows we performed a global gene expression analysis of liver tissue sampled at different time points before and after intra-mammary (IM) exposure to *E. coli *lipopolysaccharide (LPS) treatment.

**Results:**

Approximately 20% target transcripts were differentially expressed and eight co-expression clusters were identified. Each cluster had a unique time-dependent expression profile and consisted of genes involved in different biological processes. Our findings suggest that APR in the liver is triggered by the activation of signaling pathways that are involved with common and hepatic-specific transcription factors and pro-inflammatory cytokines. These mediators in turn stimulated or repressed the expression of genes encoding acute phase proteins (APP), collectins, complement components, chemokines, cell adhesion molecules and key metabolic enzymes during the APR. Hormones, anti-inflammatory and other hypothalamus-pituitary-adrenal axis (HPAA) linked mediators also seemed to participate in APR.

**Conclusion:**

Performing global gene expression analysis on liver tissue from IM LPS treated cows verified that the liver plays a major role in the APR of *E. coli *mastitis, and that the bovine hepatic APR follows the same pattern as other mammals when they are challenged with LPS. Our work presents the first insight into the dynamic changes in gene expression in the liver that influences the induction, kinetics and clinical outcome of the APR in dairy cows.

## Background

Mastitis caused by *Escherichia coli *(*E. coli*) is a common disease in lactating dairy cows. During infection, lipopolysaccharide (LPS) released from the cell wall of *E. coli *rapidly induces a sophisticated inflammatory response. Locally it is characterized by recruiting leukocytes especially neutrophils to the injured mammary tissue, as well as activation of macrophages in the mammary gland to produce pro-inflammatory cytokines [1]. These pro-inflammatory cytokines include tumor necrosis factor-alpha (TNF-α), interleukin-1 (IL-1) and interleukin-6 (IL-6), which have profound roles in the inflammatory response. Systemic consequences are observed as the acute phase response (APR) phenomena such as fever, leukocytosis and altered plasma concentration of acute phase proteins (APP). LPS is a highly potent activator of the pro-inflammatory response during *E. coli *infection and is often used to simulate the gram negative mastitis. It provokes secretion of above pro-inflammatory cytokines which in turn activate or suppress expression of acute phase genes in hepatocytes, vascular endothelium and other target cells [2,3]. Although some extra-hepatic production occurs [4,5], the liver is thought to be the major contributor to the APP in the blood. APR is also commonly accompanied by a widespread change of metabolism [6].

The contribution of the liver to the circulating levels of cytokines and APP in vivo in cattle is not fully understood. In cattle, in vitro studies using Kupffer cells (liver macrophages) have shown an important contribution of liver to the cytokine production [7]. In vitro, recombinant bovine cytokines can stimulate the secretion of haptoglobin (HP) from bovine hepatocytes [8]. Furthermore, synthesis of serum amyloid A (SAA) was shown to be mainly regulated by TNF-α, IL-1, IL-6 and glucocorticoid via a network of transcription factors and antagonists [9]. Our previous study shows that liver has a great capacity to produce both pro- and anti-inflammatory cytokines and APP when dairy cows are infused LPS intra-mammary (IM) [10]. All these studies suggest a strong connection between cytokines activity and APP production in cattle during inflammation.

The biological functions of APP such as HP and SAA in the APR are only partly known. Although the APP is highly conserved during evolution [11], their presence and importance differ among animal species. The significantly elevated APP expression in liver and increased concentration in blood and milk during udder infection indicate a crucial role of these proteins in the host's anti-pathogen response to mastitis [10,12,13]. Other genes involved in the energy metabolism, integrity and function of hepatocytes are also expected to be regulated in the liver during the APR, and may influence the induction, kinetics and clinical outcome of the APR to LPS induced inflammation in dairy cows [3].

To gain a better insight into the genes and pathways involved in hepatic APR in dairy cows, we performed a global gene expression analysis of liver tissue sampled at different time points relative to intra-mammary exposure to LPS treatment.

## Results

**Figure 1:**
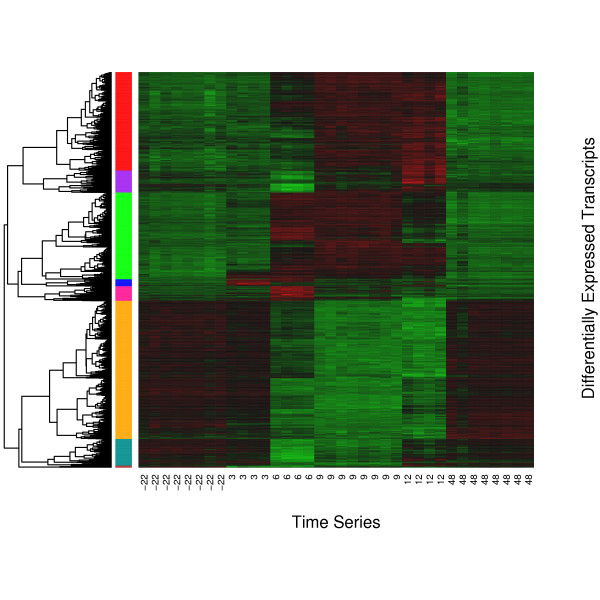
**Heatmap of differentially expressed transcript sets**. 4610 transcripts were differentially expressed (adjusted *P *< 0.01). Normalized intensity values of transcripts (rows) are ordered using Centered Pearson correlation and hierarchical clustering. The dendrogram show the similarity (distance) of expression profile of transcripts and is divided into 8 sub-trees as indicated in the color bar. Arrays (columns) are grouped according to the sampling time which varies from -22 to 48 hours subject to LPS infusion. Red and green colors reflect the high and low intensities respectively.

**Figure 2:**
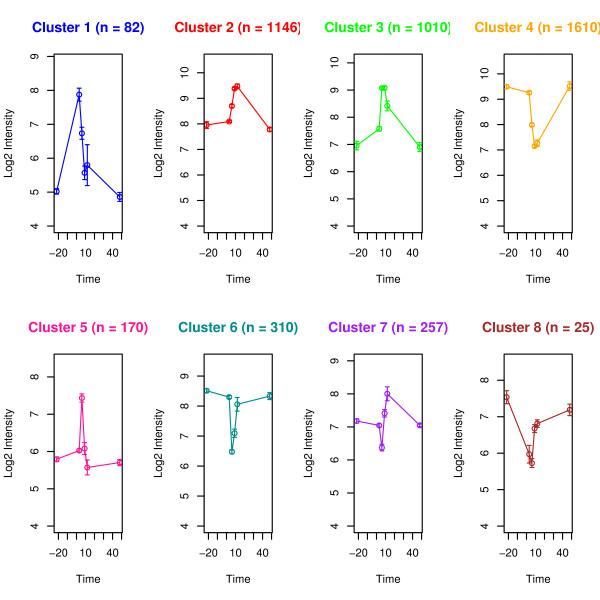
**Expression profile of individual cluster**. Each expression profile corresponds to a sub-tree (color match) in Figure 1. The cluster size (number of transcripts) is shown inside parenthesis. Y-axis represents the average value of log2-transformed expression intensity and x-axis represents the time series chronologically (-22, 3, 6, 9, 12, 48) subject to LPS treatment. A description of the genes included in each clusters are given in the result section.

Of the 24128 target transcripts measured by the microarray approximately 20% were differentially expressed (DE) at one or more time points relative to LPS treatment. A heatmap of the 4610 DE transcripts is shown in Figure 1. These transcripts were grouped into eight clusters and the average expression profile of each cluster is shown in Figure 2. Most transcripts exhibit a single upward or downward wave with the expression of 2408 transcripts increased (cluster 1, 2, 3 and 5) and 1945 transcripts decreased (cluster 4, 6 and 8), respectively, over the time course measured. However some transcripts showed a bimodal expression pattern between 6 and 12 hours after LPS treatment (cluster 7, n = 257). Transcripts from clusters 1 and 8 responded rapidly within 3 hours after LPS treatment. The majority of transcripts exhibited a maximal deviation from baseline at 6–12 hours post LPS infusion (cluster 2, 3, 4, 5, and 6). Most transcripts returned to baseline at 48 hours after LPS treatment.

**Figure 3:**
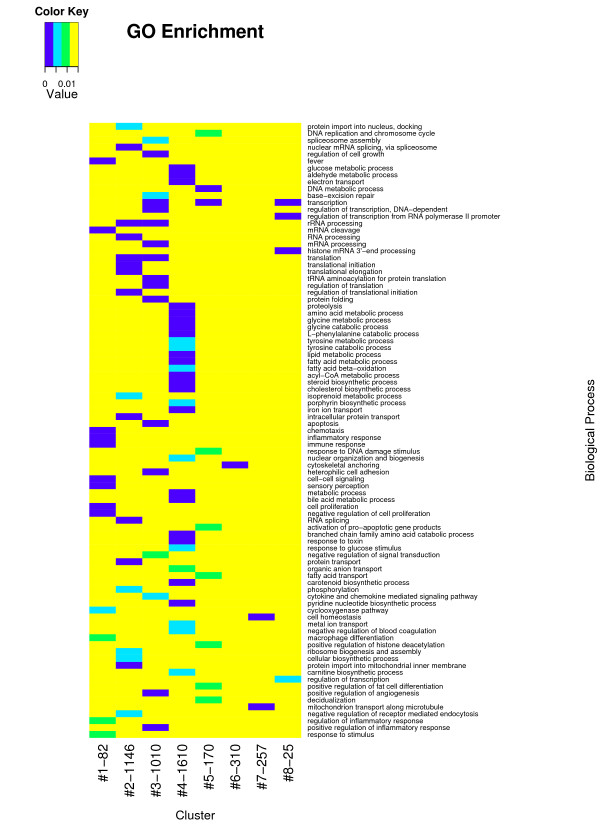
**Gene Ontology enrichment in individual cluster**. In total 89 GO biological processes (rows) are overrepresented (*p *< 0.01) within the 8 clusters. Each cluster (columns) corresponds to a sub-tree mapped in Figure 1, with the expression profile and size illustrated in Figure 2. Referring to the scale of color key, each lane stands for the *P *value of overrepresented GO-BP within each cluster. The yellow background stands for the non-significance (*p *> 0.01).

Overrepresentation of gene sets defined by the Biological Process (BP) of Gene Ontology (GO) was tested within each cluster. The BPs shown in Figure 3 was overrepresented in at least one cluster (*p *< 0.01). **Cluster 1 **contains 82 transcripts and the average expression was characterized by a rapid increase within 3 hours after LPS treatment that is followed by a quick return to the baseline (cluster 1 in Figure 2). A significant proportion of the genes in this cluster are involved in immune and inflammatory responses (GO:0006954; GO:0006955), chemotaxis (GO:0006935), cyclooxygenase pathway (GO:0019371) and macrophage differentiation (GO:0030225). It includes genes encoding IL-1 alpha and beta, chemokine (C-X-X motif) ligand 1 (CXCL1), chemokine (C-C motif) ligand 2 (CCL2), ligand 8 (CCL8), prostaglandin-endoperoxide synthase 2 (PTGS2, also known as cyclooxygenase-2, COX-2), colony stimulating factor 1 (macrophage, CSF1) and CCAAT/enhancer binding protein delta (CEBPD). **Cluster 2 **contains 1146 transcripts whose maximum expression occurred at 9–12 hours (cluster 2 in Figure 2). The overrepresented BP are phosphorylation (GO:0016310) and negative regulation of receptor mediated endocytosis (GO:0048261). This cluster includes toll interacting protein (TOLLIP), CD97, epidermal growth-factor receptor (EGFR), signal transducer and activator of transcription 3 (STAT3), AMP-activated protein kinase beta 1 non-catalytic subunit (PRKAB1) as well as HP. **Cluster 3 **contains 1010 transcripts whose expression increased 3 hours after LPS infusion and have a maximum expression at 6 to 9 hours (cluster 3 in Figure 2). Involved BP are cytokine and chemokine mediated signaling pathway (GO:0019221), positive regulation of inflammatory response (GO:0050729), negative regulation of signal transduction (GO:0009968), heterophilic cell adhesion (GO:0007157) and apoptosis (GO:0006915). The DE transcripts include the genes encoding toll-like receptor 2 (TLR2), tumor necrosis factor receptor superfamily, member 1A (TNFRSF1A), member 5 TNFRSF5, interleukin 1 receptor antagonist (IL1RN), myeloid differentiation primary response gene 88 (*MYD88*), NF-kappaB (NFKB) transcription factor p65 subunit (human homolog NFKB3), NF-kappaB inhibitor alpha (NFKBIA), activating protein-1 AP1 (also known as JUN), suppressor of cytokine signaling 3 (SOCS3) and APP SAA3 and alpha-1 acid glycoprotein (AGP) as well as intercellular adhesion molecule 1 (CD54 or ICAM-1), peroxisome proliferator activated receptor gamma coactivator 1 alpha (PPARGC1A/PGC-1/PGC-1α) and scavenger receptor class B, member 1 (SCARB1). **Cluster4 **contains 1610 transcripts whose expression was down regulated with a minimum at 9 to 12 hours after LPS administration (cluster 4 in Figure 2). The enriched biological processes are related to the amino acid metabolic process (GO:0006520), glucose metabolic process (GO:0006006), lipid metabolic process (GO:0006629), fatty acid metabolic process (GO:0006631), steroid biosynthetic process (GO:0006694) and proteolysis (GO:0006508). Among the genes in this cluster are phosphoenolpyruvate carboxykinase 1 (PCK1), glucose-6-phosphatase catalytic subunit (G6PC), corticosteroid-binding globulin (CBG) precursor and prolactin receptor (PRLR), as well as complement component 4-binding protein, beta (C4BPB) and mannose-binding lectin (MBL). **Cluster 5 **contains 170 transcripts which were up regulated temporarily from 3 to 6 hours (cluster 5 in Figure 2) and return to the baseline within 12 hours. The involved biological processes are response to DNA damage stimulus (GO:0006974), activation of pro-apoptotic gene products (GO:0008633) and DNA metabolism (GO:0006259). The DE transcripts include the gene encoding chemokine (C-C motif) ligand (CCL20). **Cluster 6 **contains 310 transcripts which were down regulated temporarily between 3 to 6 hours and returned to the baseline within 12 hours (cluster 6 in Figure 2). Cytoskeletal anchoring (GO:0007016) was found overrepresented and this cluster includes genes encoding 6-phosphofructo-2-kinase (PFKFB2), suppressor of cytokine signaling 2 (SOCS2) and hepatocyte nuclear factor 3 (HNF-3) alpha. **Cluster 7 **contains 257 transcripts. The expression profile of these genes is bimodal and is characterized by a decrease from 3 to 6 hours followed by a rapid increase from 6 to 12 hours and then returning to baseline at 48 hours (cluster 7 in Figure 2). Genes involved in cellular homeostasis (GO:0019725) and mitochondrial transport along microtubule (GO:0047497) are overrepresented. **Cluster 8 **contained 25 transcripts which rapidly declined within 3 hours (cluster 8 in Figure 2). Regulation of transcription by RNA polymerase II promoter (GO:0006357) and regulation of transcription (GO:0045449) are the overrepresented biological processes and it includes CCAAT/enhancer binding protein alpha (CEBPA).

**Figure 4:**
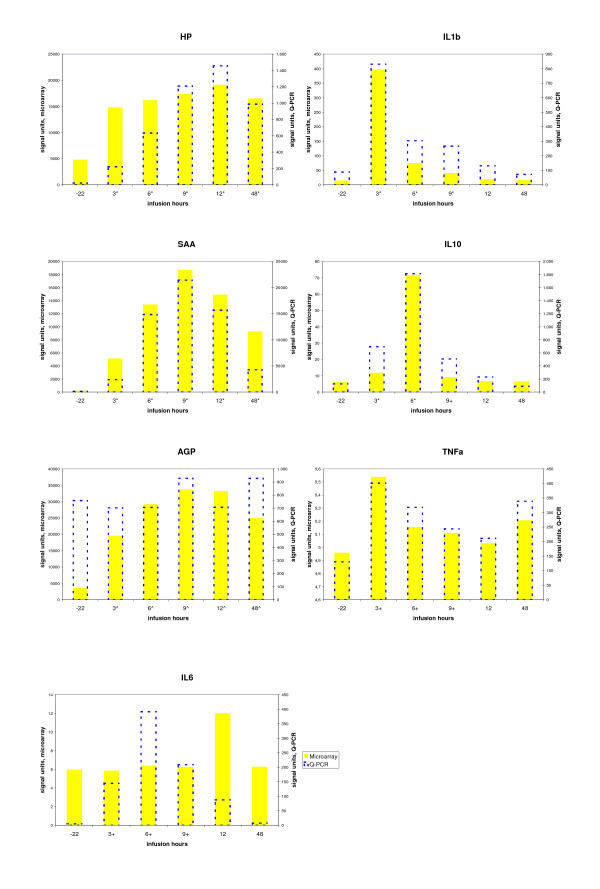
**Comparing the expression profile of 7 selected genes measured using Q-PCR and microarray**. The mRNA expression of *HP*, *SAA3*, *AGP*, *IL-1β*, *IL-6 *and *IL-10 *and *TNF-α *were detected using Q-PCR (blue unfilled bar) and microarray (yellow solid bar) technologies. The height of the bar indicates the average signal units. Left (right) Y-axis is the scale of microarray (Q-PCR) data. Asterisk (*), caret (^) and plus (+) symbol in the bottom indicate significant (p < 0.05) DE when comparing a time point after LPS treatment (3, 6, 9, 12 or 48 hours) to -22 hours before the infusion. Asterisk indicates significance in both microarray and Q-PCR; caret and plus indicate significance only in microarray or Q-PCR respectively. The signal units for microarray are the intensities before the log base 2 scale transform and the asterisk and caret signs for microarray are the p values after Bonferroni correction.

The performance and sensitivity of the microarray analysis was validated against quantitative PCR (Q-PCR) performed on the same liver tissue samples [10]. Seven inflammatory markers including the pro- and anti-inflammatory cytokine TNF-α, IL-1β, IL-6 and IL-10 and the APP HP, SAA3 and AGP were tested. All markers, except AGP were found to be significantly up regulated when measured using Q-PCR [10]. The expression profiles from the microarray showed the same trends as Q-PCR for most investigated markers except AGP and IL-6 (Figure 4). Among the cytokines IL-1β and IL-10 were significantly up regulated on the microarray, but not TNF-α and IL-6. To ensure a low false discovery rate we have chosen to adjust for multiple testing using the stringent Bonferroni method. This means that some genes with significantly low raw p-values which is the case for TNF-α and IL-6 will be deemed non differentially expressed after adjusting for multiple testing although the expression profile of TNF-α is very similar to that observed by Q-PCR. Also the two technologies have different sensitivity, because Q-PCR has a 40-cycle amplification while the Affymetrix array is loaded with cRNA that is amplified normally no more than 2 cycles. This means that inflammatory cytokine which normally are present in picogram or nanogram in blood and liver are easier detected using Q-PCR. In contrast, the APP that often are present in microgram or milligram in the blood of diseased individuals, were all found to be significantly up regulated on the microarray.

## Discussion

This study presents the first global gene expression profiling of the hepatic APR in dairy cows following intra-mammary exposure to LPS. Clinical findings (e.g. fever, high somatic cell count (SCC) in the milk, temporary leukopenia, leukocytosis) verified the induction of mastitis inflammation by LPS. The bovine array data strongly support that the hepatic APR is triggered by the activation of the signaling pathways that are involved with common and hepatic-specific transcription factors and pro-inflammatory cytokines. These mediators in turn stimulates or represses the expression of genes encoding APP, collectins, complement components, chemokines, cell adhesion molecules and key metabolic enzymes during APR. Hormones, anti-inflammatory and hypothalamus-pituitary-adrenal axis (HPAA) linked mediators also seem to participate in the modification of APR. The alteration of these acute phase genes is thought to be responsible for the observed systemic APR phenomena.

The overall gene expression pattern of the hepatic APR in dairy cows depicts what has been found in other animal LPS models such as mice, canine and sheep using different LPS application routes. In the liver of mice given intraperitoneal (i.p.) LPS there was an up regulation of genes involved in defense and immunity, and intracellular signaling while genes involved in metabolism of fatty acid, bile acid and cholesterol were down regulated [3]. Similar findings in the liver of intravenous (i.v.) LPS-challenged sheep [14,15] and canine [16] suggests that the changes in the gene expression we have in the bovine liver during APR is a common response in mammalian species. In the following sections, changes in individual gene expressions in LPS challenged dairy cows will be discussed in relation to the existing and general knowledge of the hepatic APR.

### Initiation of APR in liver

LPS IM infusion induces systemic APR, which in the liver is associated with the pro-inflammatory cytokines produced by the activated macrophages. CD14 and TLR4 are the cell membrane proteins which bind LPS and may trigger the signaling pathways of macrophages activation [17,18]. TLR2 also has a recognizable role in the inflammatory activation of liver shown in mice by i.p. LPS treatment [19]. *TLR2-MYD88 *elicits the signaling cascades that activate *AP1 *and *NFKB *[20]. The latter promotes the transcription of *IL-6 *and *TNF-α *[21,22]. We observed that the expressions of *TLR2*, *MYD88*, *AP1 *and p65 subunit (*NFKB3*) of NFKB complex, its inhibitor (*NFKBIA*), both *IL-1α *and *IL-1β*, and IL-1 receptor antagonist *IL1RN *were all up regulated. Although the increased expression of *IL-6 *and *TNF-α *is less dramatic when measured using the microarray, it is significant using Q-PCR [10]. Increased expression was also observed in the cytokine receptors like *TNFRSF1A *and *TNFRSF5 *(*CD40*), and the component of receptor pathway such as *TOLLIP*, which is an essential element of the IL-1 receptor signaling pathway [23].

Hormones are another family of key mediators involved in APR. We found a decreased expression of prolactin receptor (*PRLR*), similar to that shown in hepatic tissue after i.p. LPS injection [24]. PRL induced *STAT3 *[25] which however was up regulated in our study. One explanation is that in the liver *STAT3 *is also inducible by growth hormone (GH), IL-6 family cytokines and epidermal growth factor (EGF) [26,27], and we did observe increased expression of the EGF receptor. Activated *STAT3 *can enhance the transcription of *C/EBPs *and *AP1*, which both are important transcription factors controlling the production of APP and other inflammatory genes such as *SCARB1 *that participates in the clearance of LPS in the liver [28-30]. We observed an elevated expression of *C/EBPδ*, *AP1 *and *SCARB1*. In addition, *SOCS2 *and *SOCS3*, the attenuator of *STAT3 *mediated signal transduction, were also altered.

It seems APR predominantly relies on the activation of signaling pathways involved with transcription factors and pro-inflammatory cytokines. And it may subject to the modification by the hormones via the corresponding receptors. The coordination of pro- and anti-inflammatory mediators such as inhibitors or antagonists also participated and may provide the host with a state of "high alert" in which the immune response can be efficiently initiated if the infection progresses, and restrained if the infection is resolved or becomes excessive or prolonged.

### HPAA and APR in liver

Fever was observed in the cows after LPS challenge and as a physiological stress, it can lead to the change of serum cortisol levels. Prostaglandin (PG) E2 has a profound role in thermoregulation during infection [31]. In LPS-induced fever, first febrile phase (~0.5 hour post LPS) is triggered by the activation of PGE2 synthesis mainly in macrophages in liver and lung [32]. PGE2 is synthesized via the cyclooxygenase pathway in which COX-2 is the key enzyme. We found increased expression of *COX-2 *in the liver within 3 hours after LPS IM infusion. In fact, APR represents a cross-talk between the peripheral inflammation and central nervous system [33]. During infection HPAA is activated by pro-inflammatory cytokines and influences the inflammation by releasing glucocorticoids such as cortisol [15,34]. The majority of cortisol in serum is bound to CBG and is biological inactive [35]. We observed a decreased expression of *CBG *in the bovine liver and studies of human HepG2 cell in vitro showed that pro-inflammatory cytokines IL-1 and IL-6 can decrease CBG synthesis [36]. The expected outcome is increased levels of unbound (active) cortisol in serum in response to the local inflammation in the mammary gland. It suggests that HPAA linked mediators (cortisol and PGE2) may respond to the expression change of acute phase genes in liver.

### Expression of APP

APP is one of the most important products of the liver during the infection. We observed that the expression of major positive APP such as *SAA3*, *HP *and *AGP *were all up regulated. No significant change was observed in C-reactive protein, shown to be less responsive in cattle as compared to human, pigs and dogs during the acute infection [4]. SAA, HP and AGP are class I APP which dependent on *IL-1-NFKB *signaling pathway and contain C/EBP binding sites in their promoter regions [37]. In our study *C/EBPδ *was elevated while *C/EBPα *was decreased less than 3 hours after LPS treatment confirming the inverse relationship between these transcription factors during APR [38]. *STAT3*, a class II acute phase gene (induced by *IL-6*), was also up regulated is known to stimulate the transcription of *C/EBPs *as described previously and thereby affecting expression of the APP indirectly. The increased expression of common (*NFKB*, *STAT3*) and hepatic-selective (*C/EBPδ*) transcription factors suggests that the expression of APP is controlled by a network of transcription factors involving both classes of the acute phase genes.

It is also notable that some APP from the complement pathway or collectin family, for example *MBL*, was down regulated. Studies in humans have shown that IL-1 can suppress the *MBL *expression [39]. Another example is C4BPB, a crucial regulator in both the classical and lectin pathways. Expression levels of up stream regulators of *C4BPB *such as *HNF-3 *and *PFKFB2 *[40,41] were suppressed, which may explain the decreased expression of *C4BPB *we observed in the bovine liver during the APR. Complement components (CC) and collectins are considered as the first line of defense against pathogen invasion. However in our study, LPS was infused into the mammary gland and decreased expression of collectins and CC may be due to the absence of direct binding to the foreign surface antigen.

### Leukocytes recruitment and activation

Leukocytosis was observed in the LPS treated cows and correspondingly we also observed an increased expression of genes, which play a key role in leukocytes migration and activation in the liver. Among those genes are 1) chemokines: macrophage inflammatory protein (MIP-3α/CCL20), monocyte chemotactic proteins (MCP-1/CCL2, MCP-2/CCL8) and neutrophil-activating protein NAP-3/CXCL1; 2) cell-adhesion molecules (CAM): ICAM-1; and 3) other cell surface molecules and colony stimulating factors: CD97 and CSF-1. CD97 plays an essential role in migration of leukocytes particularly neutrophils [42], and it is a marker of early lymphocyte activation [43,44]. CSF-1 is central to the regulation of production, maintenance and function of macrophages [45]. *NFKB *and *MYD88*, which are responsible for inducing the expression of many of the above mentioned chemokines and CAM [46-50], were also up regulated suggesting their crucial role in hepatic leukocytes migration and activation.

### Metabolism and APR

A large group of metabolism genes were down regulated in the bovine liver in response to IM LPS infusion. Of particular interest was the increased expression of *PRKAB1*, a regulatory subunit of AMP-activated protein kinase (AMPK), as recent findings show that AMPK plays a major role in the control of hepatic metabolism [51]. Activation of *AMPK *leads to inhibition of glucose production and we did indeed observe decreased expression of the gluconeogenic key enzymes *PCK1 *and *G6PC *and their transcriptional co-activator *PGC-1α *[52]. Moreover, down regulation of the expression of *PCK1 *and *G6PC *by *STAT3 *[53] whose expression levels was increased in our study, indicates that the hepatic metabolism may also be influenced by transcription factors from the signaling pathways in APR. Signaling gene networks were also shown to be coordinately regulated in the liver of periparturient cows in response to ketosis [54]. In general our results does support the findings that the inflammatory response affects metabolism in dairy cows [55].

## Conclusion

Performing global gene expression analysis on liver tissue from IM LPS treated cows verified that the liver plays a major role in the APR of *E. coli *mastitis, and that the bovine hepatic APR follows the same pattern as other mammals when they are challenged with LPS. Our work presents the first insight into the dynamic changes in gene expression in the liver that influences the induction, kinetics and clinical outcome of the APR in dairy cows.

## Methods

### Animals and LPS treatment

The experimental design has previously been reported [10]. In short eight healthy, high yielding (38 kg milk per day) Holstein-Friesian dairy cows in their first lactation (9 to 12 weeks after calving) were chosen for this study. The udder health of the cows was evaluated based on somatic cell count (SCC) and bacteriological examinations. All cows had SCC < 100,000 on both front quarters and SCC < 138,000 on both behind quarters, and were free from mastitis pathogens. Sterile polyvinyl catheters (Micro-Renathane) were inserted into the jugular vein of the cows. The catheters were flushed with a sterile 0.9% NaCl solution containing 50 IU/ml Na-heparin (Loevens Kemiske Fabrik, Ballerup, Denmark). At time 0 the right front quarter was infused with 200 μg *E. coli *LPS (0111:B4) (Sigma-Aldrich, Brøndby, Denmark) dissolved in 10 ml 0.9% NaCl solution, the left front quarter serving as control was infused with 10 ml 0.9% NaCl solution. Production data, clinical signs as well as blood and milk parameters associate with LPS treatment were recorded throughout the experiment. The clinical and paraclinical findings verified the induction of mastitis inflammation by LPS. LPS intra-mammary infusion induced fever and a high SCC in the milk of the treated quarter. Further, increased concentrations of TNF-α, SAA, HP were found simultaneously with temporary leukopenia followed by leukocytosis and all characteristic observations in an LPS IM infusion model. Quantitative PCR also confirmed the high expression of cytokines *TNF-α*, *IL-1β*, *IL-6 *and *IL-10 *and the acute phase proteins *SAA3 *and *HP *in the liver following IM LPS infusion [10].

All procedures involving animals were approved by the Danish Animal Experiments Inspectorate and complied with the Danish Ministry of Justice Laws concerning animal experimentation and care of experimental animals.

### Liver biopsies

Liver biopsies were taken at -22, 3, 6, 9, 12 and 48 hours relative to LPS infusion in 4 cows, and also at -22, 9 and 48 hours in the remaining 4 cows. Sampling procedures for liver biopsies were as previously described [56]. A control study using cows infused with 0.9% NaCl showed that there was no effect of taking the biopsy, neither in the clinical measurement nor in the expression of a selected subset of genes [10]. Therefore, only 36 samples taken from the LPS treated cows were measured for the gene expression using microarrays.

### RNA purification and microarray processing

Isolation and labeling of RNA and microarray processing were performed as described elsewhere [57]. RNA from liver biopsies was isolated using Trizol Reagent (Invitrogen, Taastrup, Denmark). Five μg RNA was labeled using the SuperScript Choice System (Life Technologies) according to the manufacturer's instructions, except for using an oligo-dT primer containing a T7 RNA polymerase promoter site. Biotin labeled cRNA was prepared using the BioArray High Yield RNA Transcript Labeling Kit (Enzo, Farmingdale NY, USA). 15 μg of cRNA was loaded onto the probe array cartridge of Bovine Genome Array (Affymetrix Clara CA, USA). The array contains 24128 probe sets which represent 15264 UniGene (annotation from May, 2006) to measure the global transcripts. Hybridization mixture of each sample (a specific cow at a certain time point) was loaded to an array resulting in a total of 36 arrays.

### Annotation

Bovine Genome Array annotation is available from the NetAffx. Genes and gene products attributes are characterized with the Gene Ontology (GO). However, in NetAffx (December, 2005) only ~16% of probe sets on the array were GO annotated. Additional annotation was obtained from the Ensembl database, using the biomaRt package in R [58] as the query interface. The query IDs (Ensembl gene ID, Entrez gene ID or Affy probe set ID) were extracted from the NetAffy annotation. Besides bovine genes, we also retrieved the annotation based on gene sequences of the human ortholog in Ensembl. From above resources ~48% of the probe sets on the array were able to be annotated with GO.

### Statistical analysis

The data was analyzed using R (version 2.4.1), a programming language and development environment for statistical computing and graphics. Normalization of expression values was performed using the GeneChip Robust Multi-array Analysis (GCRMA) algorithm [59,60]. In this algorithm, raw intensity values are background corrected based on a model using sequence information followed by quantile normalization [60,61]. This algorithm combines the strength of stochastic-model based algorithms and physical models and has been shown to be superior in accuracy and precision to other normalization methods such as MAS, RMA and PerfectMatch [62]. The microarray data has been deposited in the Gene Expression Omnibus database (accession GSE10695).

Differential expression of individual genes was assessed using linear modeling and empirical Bayes methods [63] as implemented in the R package Linear Models for Microarray Analysis, LIMMA [64]. The linear models allows for general changes in gene expression between successive time points (3 to -22, 6 to 3, 9 to 6, 12 to 9 and 48 to 12). Each transcript targeted by the probe set was tested for the expression change between all pairs of the successive time points using a modified t-test, and for multiple contrasts using a moderated F test. The residual standard deviations are moderated across the probe sets to ensure more stable inference for each transcript. The moderated standard deviations are a compromise between the individual transcript-wise standard deviations and an overall pooled standard deviation. Since 24128 transcripts were measured by the array and tested for the alteration simultaneously, the multiple-tests adjustment is introduced to control the number of false positives. The multiple testing was accounted for using the Bonferroni method. Transcripts were deemed differentially expressed (DE) if one or more adjusted *P *values for the moderated t-test was below 0.01. The adjusted moderated F statistics also showed that all DE transcripts, except [GenBank: CK775003] (the probe set is Bt.21216.1.S1_at), were significant.

**Figure 5:**
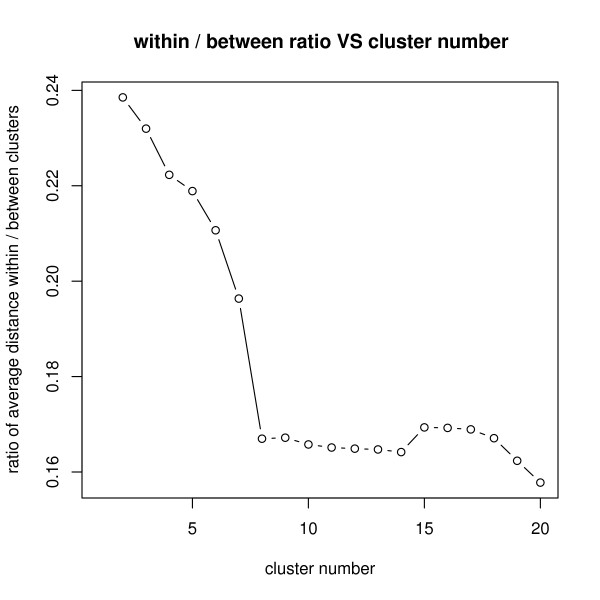
**Determine the number of clusters**. 4610 differentially expressed transcripts were hierarchically clustered using Centered Pearson correlation. The optimum cluster number (X-axis) was determined using the ratio of the sums of squares between and within cluster (Y-axis). We chose 8 clusters as it classified the transcripts into relatively few clusters while maintaining maximum similarity of expression pattern within the same cluster.

To identify co-expression clusters, the 4610 DE transcripts were ordered by the hierarchical clustering. Centered Pearson correlation method was used as the distance measure. The optimum cluster number was determined using the ratio of the sums of squares between and within cluster [65] (see Figure 5).

### Gene set enrichment

There are 1789 UniGenes possessing Gene Ontology (GO) among all DE transcripts in contrast to 6843 UniGenes from the whole array. Within each co-expression cluster, overrepresentation of gene sets defined by the Biological Process (BP) of GO was tested using Fisher's exact test.

## Abbreviations

AMPK: adenosine monophosphate-activated protein kinase; APP: acute phase proteins; APR: acute phase response; ATP: adenosine 5'-triphosphate; BP: biological process; CAM: cell-adhesion molecules; CC: complement components; cRNA: complementary ribonucleic acid; DE: differentially expressed; *E. coli*: *Escherichia coli*; EGF: epidermal growth factor; GCRMA: GeneChip robust multi-array analysis; GH: growth factor; GO: gene ontology; HPAA: hypothalamus-pituitary-adrenal axis; IM: intra-mammary; i.p.: intraperitoneal; IU: international units; i.v.: intravenous; LIMMA: linear models for microarray analysis; LPS: lipopolysaccharide; MAS: microarray suite; MCP: monocyte chemotactic protein; MIP: macrophage inflammatory protein; NAP: neutrophil-activating protein; PG: prostaglandin; PRL: prolactin; PRLR: prolactin receptor; Q-PCR: quantitative polymerase chain reaction; RMA: robust multi-array analysis; SCC: somatic cell count

## Authors' contributions

LJ and PS analyzed the microarray data and drafted the manuscript. CR carried out the animal challenge, KLI performed the liver biopsy and LV performed the Q-PCR. PS, CR and KLI designed the experimental plan. LJ, PS, CR and KLI contributed to the interpretation of results and iterative refinement of the manuscript. LJ is responsible for depositing microarray data in NCBI GEO.  LJ and PS contributed equally to this work.
